# Benthic Community Composition of Coral Reefs Is Associated with the Time Since Last Blast Fishing Event

**DOI:** 10.3390/biology15181584

**Published:** 2026-09-09

**Authors:** Alec Sullivan Leitman, Melissa Hampton-Smith, Ivor Growns, Sydney Lyn Bell, Raj Heinz Mathias

**Affiliations:** 1 College of Science and Engineering, James Cook University, Townsville, QLD 4811, Australia; sydney.bell@my.jcu.edu.au (S.L.B.); rajheinz.mathias@my.jcu.edu.au (R.H.M.); 2 College of Arts, Society and Education, James Cook University, Townsville, QLD 4811, Australia; melissa.hamptonsmith@my.jcu.edu.au; 3 School of Environmental and Rural Science, University of New England, Armidale, NSW 2351, Australia; igrowns@une.edu.au

**Keywords:** blast fishing, coral reefs, destructive fishing, benthic community composition, reef disturbance

## Abstract

Blast fishing is a widespread destructive practice that has long-term negative impacts on coral reef ecosystems. To better understand the long-term impact of blast fishing on these ecosystems within Southern Tanzania, in situ surveys were undertaken within a two-year timeframe across six reef sites in Mikindani Bay. These sites varied in the time since blast fishing was last undertaken (<5 years, 5–10 years, or >10 years). While there were limited differences in major benthic categories (soft substrate, algae, and hard coral), multivariate benthic community mix varied significantly with the time since last blast, which explained approximately 25% of the observed variation. This compositional shift was characterized by prominent transitions among major benthic genera including branching *Acropora*, *Euphyllia*, massive *Porites*, and *Xenia.* This finding suggests that community composition varies across areas with different histories of blast fishing. Coral communities at sites with a more recent history of blasting (<5 years) were largely characterized by a high relative proportion of massive *Porites,* while sites with intermediate times since blasting (5–10 years) showed a distinct dominance of *Euphyllia*. In contrast, sites with the longest time elapsed since blast fishing ceased (>10 years) were distinguished by two main taxa; *Xenia* and branching *Acropora*. These results suggest that the long-term composition of coral assemblages is highly sensitive to historical blast fishing disturbances. The effective prevention of blast fishing in Mikindani Bay, Tanzania and coral reefs more broadly is therefore critical to conserve the remaining cover, diversity, structure and biological functions of these marine habitats.

## Simple Summary

Blast fishing is a detrimental practice that has immediate and long-term negative impacts on coral reefs. To improve our understanding of how this practice impacts coral reef communities over time in Southern Tanzania, we conducted underwater surveys between 2022 and 2024 at six different sites in Mikindani Bay. Sites varied with regard to the time since blast fishing was last conducted: <5 years, 5–10 years, or >10 years. Our results show significant differences in coral community composition among these three blast ages. While the overall percent cover of major benthic groups (such as hard coral, algae, and other invertebrates) did not significantly differ, multivariate analyses revealed distinct shifts in the relative abundance and dominance of major benthic genera. This suggests that the time elapsed since the last blast fishing disturbance is associated with the trajectory of coral reef recovery through shifting community composition, rather than changes in benthic cover. Observed variation in benthic assemblages at sites subject to blast fishing suggests that ecosystems are constantly changing as a response to disturbances. This reinforces the urgent need to prevent blast fishing in Mikindani Bay, Tanzania and at other global coral reef locations.

## 1. Introduction

Coral reefs are ecologically and economically important marine ecosystems that are increasingly impacted by the combination of natural and anthropogenic disturbances, leading to sustained habitat degradation that undermines critical ecosystem goods and services [1]. Important ecosystem services that are provided by healthy or functionally intact coral reefs include coastal protection, food provisioning, and nutrient cycling [2]. In addition to these services, coral reefs act as immense economic engines, generating an annual global tourism value of $36 billion USD [3]. Despite their vital ecological role, coral reefs and other coastal ecosystems remain vulnerable to both natural disturbances (i.e., cyclones and disease outbreaks) and anthropogenic perturbations (i.e., pollution and destructive fishing) [4]. Given the immense importance of marine ecosystems and the anthropogenic disturbances that directly threaten the biodiversity, structure and function of coral reefs [5], its necessary to prevent these types of activities that undermine key ecosystem services [6].

Blast fishing (also known as dynamite fishing; [7]), is a widespread destructive fishing practice that has long-term negative impacts on marine ecosystems [7,8,9]. According to previous research, blast fishing kills fish and shatters hard coral skeletons, creating expansive unstable coral rubble fields [10]. While generally illegal and widely condemned, blast fishing continues to occur across many tropical countries, especially in Southeast Asia and East Africa [10]. Critically, blast fishing not only impacts target species, but directly affects the biological and structural complexity of reef habitats [11]. Furthermore, blast fishing can greatly extend the ecological impacts of fishery extraction, undermine fishery sustainability, impede habitat conservation efforts [12], and negatively alter processes linked to the resilience and recovery of reefs [6,13,14].

Reefs in Tanzania have been subject to blast fishing since the 1960s, with extensive blast fishing recorded in the 1980s and 1990s [15]. Blast fishing is illegal within Tanzania and results in financial and legal consequences [16]. Despite an explicit ban on blast fishing in 1970, fishery enforcement has been irregular and hampered by corruption, insufficient funding, and inadequate mitigative training [17]. Up until 1997, blast fishing was common throughout Tanzania [18]. Between 1997 and 2002, government enforcement dramatically reduced the incidence of blast fishing [15]. A subsequent decrease in enforcement efforts saw blast fishing resurfacing and returning to sustained high levels until 2016 [15,19]. Following the upheld occurrence of blast fishing in 2016, with renewed government implementation, these restored efforts saw a dramatic reduction in blast fishing that was maintained for at least the following three years [17].

Despite optimistic progress on legislative actions against blast fishing, the long-term ramifications of this fishing practice have continued to impact other global regions in addition to Eastern Africa and have been less studied in Tanzania. Specifically, Southern Asia has been an area of high prevalence of blast fishing [10], with previous studies focusing on natural coral recruitment within rubble fields and compounded impacts of these rubble fields [6,8], impacts of blast fishing operations on localities in Indonesia [20], and motives behind this destructive fishing practice in Hong Kong, Malaysia, and Philippines [21]. In East Africa, there have been studies on blast fishing focused on spatial mapping [17], economic impact/socio-economic drivers [15,22,23], and acoustic detection [24]. While numerous studies explore blast fishing across various ecological contexts, there are few long-term studies on habitat recovery following the suspension of blast fishing.

The purpose of this study was to assess variations in benthic cover and composition among different study sites in Mikindani Bay, southern Tanzania, which varied in the time since they were last subject to blast fishing. We used image analysis to quantify the percentage cover of major benthic categories. Our study aims to better understand the long-term status and composition of coral reef communities following historical destructive fishing practices, providing critical insights for reef conservation and management in Tanzania and the broader Western Indian Ocean.

## 2. Materials and Methods

Shallow subtidal areas of southern Tanzania are characterized by sand, seagrass beds, and coral patch reefs with an extensive outer forereef that extends from Ras Masangamkuu in Mikindani Bay towards the Mozambican border [25]. Coral mining has been a common practice here, which inhabitants of this region depend on for their income [26,27]. Although there is no information available about the exact location of coral extraction, coral mining has been reported in Mikindani Bay, with the most intensive mining occurring at a depth of <2 m [27]. The impacts of coral mining have resulted in an indirect loss of coastal habitats, as well as a homogenization of surface topography, and can undermine local bans on blast fishing and further hinder coral recovery [28].

### 2.1. Sampling Design

**Figure 1:**
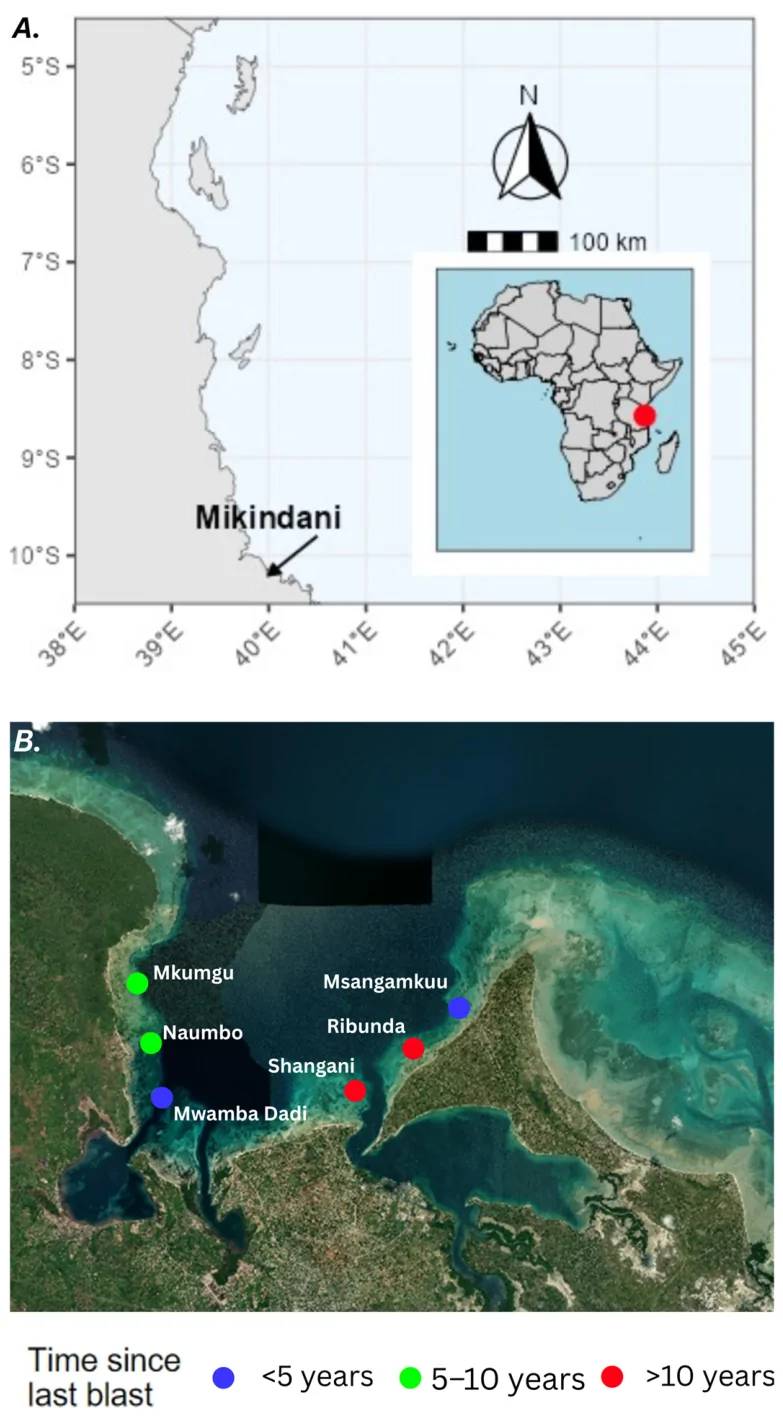
(**A**) Map of Africa, eastern coast of Tanzania and Mikindani Bay indicating site location. (**B**) Map of Mikindani Bay illustrating six reef survey sites categorized by blast age.

Data collection was conducted in Mikindani Bay in southern Tanzania, across six sites (Figure 1, Table S1). We established the minimum time since blast fishing was last undertaken at each site by consulting four local experienced fishermen and compared their responses for consistency. Informants agreed that blast fishing had been extensive across all sites. Due to the illegal and sensitive nature of blast fishing, information was obtained through informal conversation rather than structured surveys. To reassure informants, we provided information about our affiliations and research aims. No identifying data was obtained from fishermen informants. We then used this information to categorize sites to one of three distinct treatments: <5 years (*n* = 2 sites; Msangamkuu and Mwamba Dadi), 5–10 years (*n* = 2; Mkumgu and Naumbo), and >10 years (*n* = 2; Ribunda and Shangani). Field surveys were conducted during four survey periods: December 2022, July 2023, January 2024, and September 2024. Surveys were conducted at approximately 6-month intervals; some survey data collection was delayed due to staff availability and seasonal weather conditions. Two 50 m haphazardly placed photo line transects were conducted at each site during each survey period (*n* = 48), with one photo taken every meter. Benthic surveys were conducted on the upper slope side of the center line of the transect, approximately 50 cm from the tape. While images were photographed, the camera was held at a consistent distance from the substrate, approximately 20 cm, and the image area was 25 cm by 50 cm. Global Positioning System (GPS) coordinates were recorded at the beginning and end of each transect. Subsequent surveys revisited these transect lines as closely as possible. Survey depth varied according to tides but remained between six to nine meters. Reef habitat type was broadly consistent across all sites.

Image analysis was conducted using CoralNet, which is an online platform that utilizes deep learning neural networks [29]. A total of 2534 images were uploaded to CoralNet, including supplemental images taken during the survey period beyond the baseline 50 images projected per transect. Each image was annotated by selecting five random points using the simple random point generation method, covering the full frame of each photo (X: 0–100%, Y: 0–100%). Among the 48 transects undertaken across six distinct sites, we identified groups down to the genus level (Table S2).

All 2534 images, including supplemental images, were retained for CoralNet classifier training. We deployed two classifiers, which are specific models trained on the image data that were uploaded to the CoralNet platform. Annotations created by these classifiers were under a strict 100% confidence threshold (Table S1). These annotations used the default EfficientNet feature from CoralNet, which analyzes pixel patches on images uploaded onto the platform and converts this image data into numerical representations of organisms, patterns and textures [30]. To ensure stringency with the data, the classifiers’ confidence threshold was set to 100%. Since machine-calculated probabilities for annotations do not reach this absolute value, this setting disabled automatic model confirmation, which forced all machine-suggested labels to require confirmation. A single human operator verified all point annotations across all 2534 images to eliminate multi-observer bias [31]. Classifier 1 was trained on 1939 manually confirmed images, yielding a validation accuracy of 87%. After an additional 430 images were added due to supplementary survey periods in 2024, CoralNet generated classifier 2, based on the cumulative pool of 2369 images, reaching a final validation accuracy of 88%.

### 2.2. Data Analysis

A total of 48 taxonomic labels were used to categorize benthic assemblages, including hard coral genera such as branching *Acropora*, massive *Porites, Montipora*, and *Euphyllia* (Table S2). Current taxonomic research has highlighted the complexity of distinguishing coral species; therefore, all taxonomic groups utilized in analyses were limited to the genus level, which has been shown to be sufficient in assessing community composition [32,33,34]. Differences in coral assemblages based on blast age (<5 years, 5–10 years, or >10 years) and survey period were analyzed using permutational multivariate analysis of variance (PERMANOVA+) [32,35]. Treatment and survey period were treated as fixed factors, with sites nested within treatments and transects nested within sites. The response variable for analyses was absolute point cover. Bray–Curtis dissimilarity was used to create a distance matrix between samples, and probabilities were tested using 9999 permutations. Zeros and rare values were taken into account with the Bray–Curtis dissimilarity, and the variance of each source of variation was calculated according to the PERMANOVA algorithm.

The permutational multivariate analysis of dispersion (PERMDISP) test function in the PERMANOVA+ 1 for PRIMER (version 7.0.25) software was used to assess differences in sample dispersion within each blast age [33]. Differences in coral assemblages and dispersion between sites within treatments were visualized using the bootstrap average algorithm for metric multidimensional scaling ordination (MDS), with the data pooled for each transect. Additionally, the similarity percentage (SIMPER) algorithm in the PERMANOVA+ software identified taxa contributing to the benthic dissimilarities between blast ages. Differences in percent cover of major groups, including all biota, hard coral, algae, and other invertebrates, taxonomic richness and Shannon diversity between blast ages were tested with the same statistical model as for coral assemblages, using Euclidean distance and 9999 permutations.

## 3. Results

### 3.1. Major Benthic Categories

**Figure 2:**
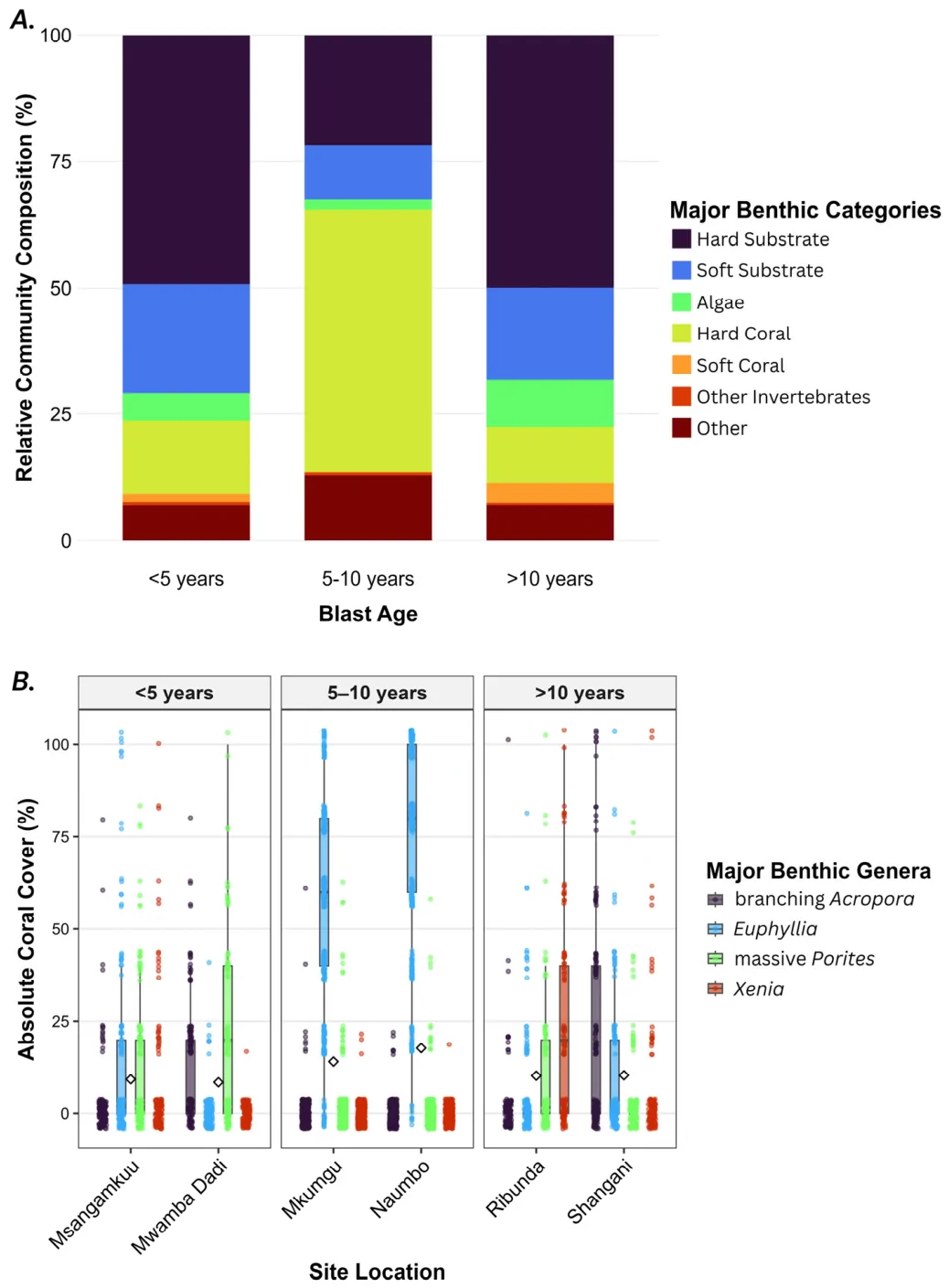
(**A**) Major benthic categories by relative community composition (%) across three blast-age treatments (<5 years, 5–10 years, and >10 years). (**B**) Absolute coral cover (%) of four major benthic genera (branching *Acropora*, *Euphyllia*, massive *Porites*, and *Xenia*) with sites nested within blast-age treatments. Raw data points per image are plotted to show variation among sites, and white diamonds denote average coral cover per site.

The relative cover of major benthic categories was consistent among sites in Mikindani Bay (Figure 2A). Despite a visible spike in the relative abundance of hard coral, mainly consisting of *Euphyllia* within the 5–10 years blast age, univariate analyses revealed no significant effect of blast age on the absolute cover of major benthic groups (pseudo-F_2,3_ = 42.9, *p* = 0.133) (Figure 2, Table S3).

### 3.2. Benthic Community Composition

While there were limited differences in the absolute cover of major benthic categories (i.e., hard coral, soft substrate, algae, etc., Figure 2A), multivariate analyses revealed that benthic community composition varied significantly by blast age (PERMANOVA, *p* = 0.0231). Blast age explained approximately 25% of the observed variation (Table 1, Figure 2B). In contrast, spatial and temporal factors, including survey period, site, transect, and their respective interactions, accounted for smaller proportions of variance (≤6%) and were not considered further.

**Figure 3:**
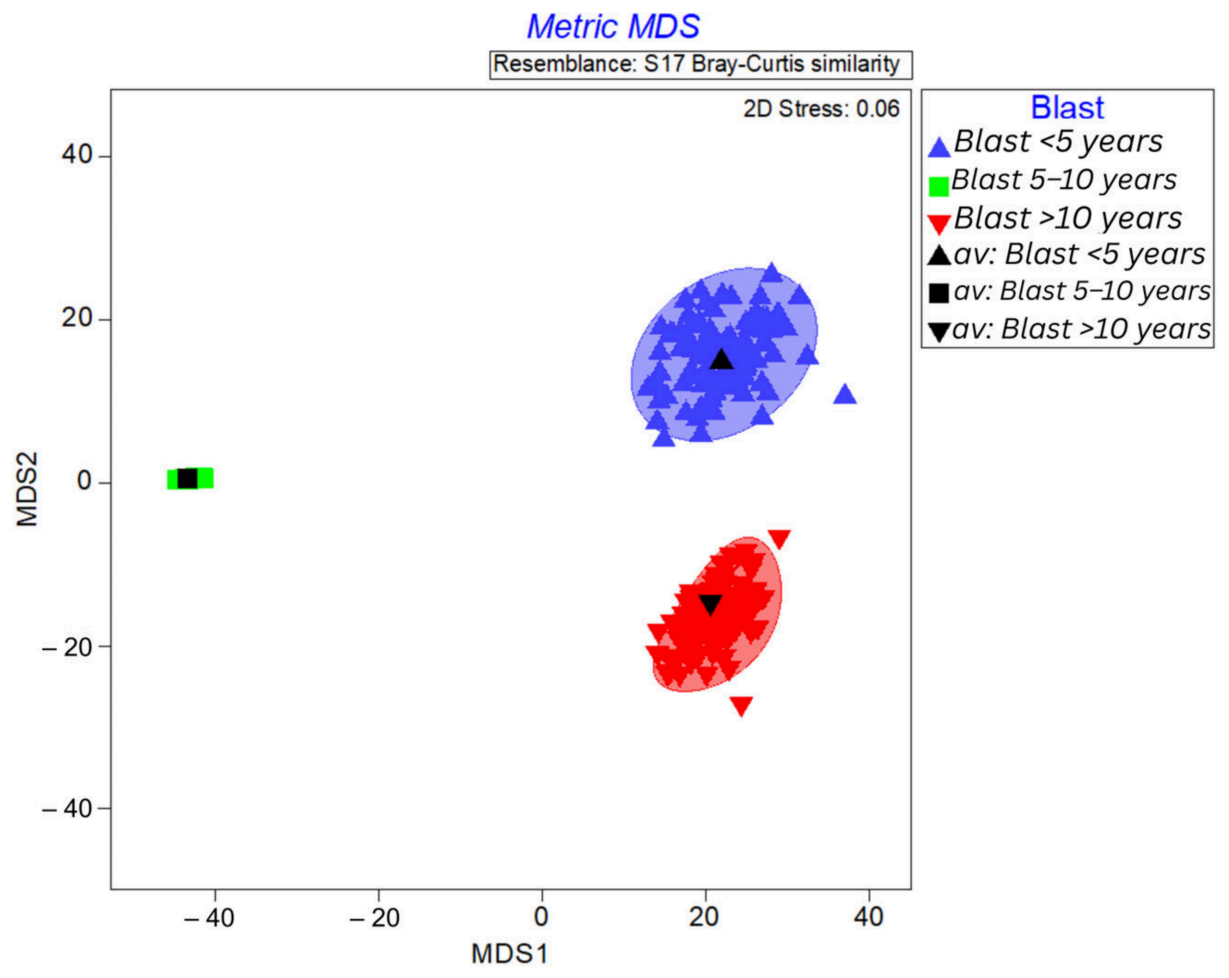
Metric multidimensional scaling (MDS) ordination plot based on Bray–Curtis similarity of major benthic genera across sites. Sites were categorized by blast age: <5 years (blue triangles), 5–10 years (green squares), and >10 years (red inverted triangles). Black symbols denote group centroids (average community composition), and ellipses indicate 95% confidence intervals around the centroids.

Coral assemblages in each of the three treatments (<5 years, 5–10 years, and >10 years) were distinct and did not overlap in multivariate space (Figure 3). Coral assemblages at sites where blast fishing occurred <5 years were characterized by a dominance of massive *Porites*, while coral assemblages at intermediate sites (5–10 years) had a high relative abundance of *Euphyllia*. Coral assemblages at sites where blast fishing occurred >10 years ago were characterized by a high relative contribution of branching *Acropora* and *Xenia* (Figure 3, Table S4).

Aside from differences in coral composition, there were also significant differences in dispersion among treatments (PERMDISP, *p* = 0.001, Table 2). Because dispersion differs strongly among the blast-age categories, the significant main effects in the PERMANOVA must be interpreted with caution, as the test cannot entirely decouple changes in community composition (site) from changes in within-group variance (dispersion). Nonetheless, clear centroid separation was observed in the MDS ordination plot (Figure 3), accompanied by high average dissimilarity amongst blast-age categories (SIMPER, <5 years vs. 5–10 years = 92.30, <5 years vs. >10 years = 89.76 and 5–10 years vs. >10 years = 91.20, Table S4). Sites that were subject to blast fishing <5 years ago exhibited less clustering and higher variability in community composition, while sites that were monitored 5–10 years post-blast exhibited tighter clustering and less variability in community composition. Thus, the significant PERMANOVA results coincide with both distinct visual grouping on the MDS and significant differences in within-group variance.

## 4. Discussion

This study has revealed marked shifts in multivariate benthic community composition among sites within Mikindani Bay, despite a lack of significant differences in the absolute cover of major benthic categories. While these compositional patterns are interpreted to be a result of blast fishing, Tanzanian reefs face other local disturbances that could indirectly act as potential confounding factors, including sedimentation from shipping ports [34,36] and the overfishing of keystone species such as the red-lined triggerfish [27]. Despite the potential influence of these confounders, Mikindani Bay’s conservation status and relatively low localized anthropogenic impacts [37] likely mitigate these broader disturbances. Spatial variation in coral composition may also relate to the specific location and proximity of individual sites (discussed below). Notably, sites with the longest time elapsed since a blasting event were characterized by structurally fragile taxa (e.g., branching *Acropora*). Such fragile taxa are likely disproportionately impacted by blast fishing compared to competing coral genera that exhibit a greater capacity to withstand physical disturbances, such as massive *Porites* [38,39]. Because we lack baseline data prior to the cessation of blasting, directly tracking specific successional trajectories remains difficult. Nonetheless, our findings support the previous literature demonstrating that blast fishing has profound, lasting impacts on coral assemblages [38,39]. While the majority of the blast fishing literature indicates that reefs rehabilitation may only be possible with active human intervention [40,41], our findings suggest that either long-term natural community transitions can occur over time, or the localized extent of historical blast damage did not permanently destabilize the benthic system.

Three main taxa, massive *Porites*, *Euphyllia*, and *Xenia*, were identified to be key drivers of compositional differences in reefs affected by blast fishing, while branching *Acropora* was an additional taxon driving these differences. Given the documented functional traits of massive *Porites*, *Xenia*, and branching *Acropora*, they can potentially serve as indicators of coral community succession over time in Mikindani Bay. Rubble fields from blast fishing [6] or other disturbances [42] are typically colonized first by pioneer binders (e.g., turfs and macroalgae) then intermediate binders (e.g., soft corals, sponges, and bryozoans) [43]. While the replacement of intermediate binders with Scleractinia is expected with improved reef conditions, this is not guaranteed, and soft coral dominance has been shown to persist for years to decades [44,45]. Furthermore, in regions such as Southeast Asia, destructive fishing has severely impeded coral recovery due to unstable rubble fields [8], and such a substrate has induced high mortality amongst coral recruits, stalling natural recovery for decades.

At the earliest blast-age sites, benthic communities were characterized by a dominance of massive *Porites.* This genus has been shown to exhibit reduced fragmentation under natural disturbances (i.e., typhoons) [46], as well as physical storm and wave conditions [47]. Indeed, a quantitative trait-based approach to classifying coral taxa characterized massive *Porites* species in the Indo-Pacific as stress-tolerant due to their longer generation times, high fecundity, and energy storage [48,49]. Furthermore, the dominance of *Porites* was also observed in Indonesia following blast fishing, acting as the predominant genus of hard coral contributing to rubble binding and accounting for the most coral recruits [50]. Altogether, this robustness against physical disturbances combined with advantageous traits in harsh environments (i.e., increased sedimentation) can potentially provide an advantage to massive *Porites* during blasts, compared to other scleractinian corals, and could be a reason for the high relative abundance of massive *Porites* in <5 year blast ages.

In our results, we found that *Euphyllia* characterized sites 5–10 years post-blast fishing. *Euphyllia* has not been shown to be a common taxon associated with successional reef recovery [51], and instead their localized abundance in 5–10 year blast ages could be attributed to on-site conditions within Mikindani Bay. The semi-enclosed nature of Mikindani Bay has potentially buffered it from anthropogenic impacts and turbidity [52]. However, the west side of the bay near Naumba, where sites 5–10 years post-blast were, may be more sheltered from wave action than the rest of the bay, providing low-energy waters where *Euphyllia* can thrive without physical damage [53]. This area also likely acts as a deposition zone as wave action is too weak to keep particles suspended. Combined with terrestrial runoff from nearby Naumba, the west side of Mikindani Bay may become the perfect environment for a large-polyp coral that is highly efficient in using a mucus coat to shed falling silt [54]. Together, these hypotheses may explain why *Euphyllia* was dominant at both intermediate blast-age sites.

Communities in >10 years blast-age sites were co-dominated by *Xenia* and branching *Acropora.* Soft corals, such as *Xenia*, can inhibit hard corals from settling [55] and directly kill neighboring colonies due to defensive chemical compounds known as allelochemicals [56]. Despite these chemicals being used for defensive purposes, Acroporid corals have been shown to successfully colonize areas in the presence of *Xenia* [55], which could explain the high relative abundances of branching *Acropora* alongside *Xenia* in >10 year blast-age sites. Additionally, we hypothesize that *Xenia’s* dominance in late-stage sites could be attributed to its ability to colonize different substrates such as rubble, sand, and debris while suppressing hard coral recruitment [55]. Critically, the proliferation of soft corals such as *Xenia* could prevent the rigid binding of rubble by hard-bodied cnidarians, harming the recovery of rubble beds [43].

Early-stage communities are more susceptible to biotic processes including species recruitment and competition [57], and opportunistic taxa, such as some pocilloporids and faviids [49], can potentially rapidly dominate any available substrate [8]. Previous research has predicted that coral communities will shift towards inhabitants that exhibit stress-tolerant/plastic traits and weedy species under multiple stressors [51,58]. However, our community composition results diverge from these expected trends, suggesting that our results are specific to Mikindani Bay. Indeed, binding organisms on rubble beds have been shown to be highly influenced by a variety of environmental and ecological parameters such as water quality, sedimentation, and temperature, driving differences in community composition across locations [43,59,60]. To better understand how certain taxa respond and react to blast fishing disturbances, incorporating biological (i.e., species-level abundance and size frequency distributions) and environmental metrics (i.e., sea-surface temperature, oxygen levels, and light availability) through long-term monitoring of multivariate metrics would strengthen ecological assessments and provide tangible insights for management.

Community composition was highly variable within sites that had the most recent blast history, compared to the older blast-age categories. The high multivariate dispersion <5 years post-blasting, followed by tight clustering at 5–10 years, and intermediate variability at >10 years, highlights distinct differences in community structure across the post-disturbance timeline. As noted, in our results, this strong variance requires a conservative interpretation of our PERMANOVA analysis, as location and dispersion effects remain statistically intertwined. Mathematically and ecologically, however, this pattern reflects a shifting successional trajectory across the site categories. Blast fishing causes immediate mortality, and the subsequent loss of taxa may trigger cascading downstream effects, such as reduced resilience to natural perturbations [8]. Consequently, the high compositional variation observed <5 years post-blasting likely represents these initial high mortality rates, similar to those observed in Indonesia [8]. The high variability observed in our sites <5 years post-blast could also reflect early colonization processes and competitive interactions among benthic organisms. Conversely, the significantly tighter clustering observed at 5–10 years suggests a transitional phase where benthic community structures temporarily homogenize, before shifting toward intermediate variance at the >10 year sites. This homogenization was also found during research in Taiwan, where early settlers rapidly controlled available space following a physical disturbance, driving widespread community homogenization and a decline in diversity [61]. While this may represent a shared successional stage, it is highly likely that site-specific environmental conditions heavily influence this pattern. Specifically, if the 5–10 year sites represent highly suitable habitats for a single dominant genus like *Euphyllia,* this localized environmental preference could artificially drive down community variance and produce the observed tight clustering. Notably, the <5 year sites had the greatest physical distance between them while the 5–10 year and >10 year sites were immediately adjacent. Therefore, geographic locations potentially introduced variation into the treatment sites. Future studies would benefit from the inclusion of additional sites and transects to provide further benthic information on the community composition throughout each survey period. Furthermore, while coral rubble was included as a broad benthic label within CoralNet, it was not subjected to univariate analysis due to its low overall presence within the annotated dataset (four out of 12,670 annotated points among 2534 images). The low rubble count is potentially reflected in a methodological limitation of utilizing five annotation points per image, which would potentially omit structural features or taxa. Furthermore, coral rubble was undoubtedly present across sites; however, the low count could be attributed to increased habitat complexity, whereby physical substrates such as coral rubble could be potentially overgrown by taxa such as *Xenia* [6] and crustose coralline algae (CCA) [62]. Due to annotation points being classified by surface-layer organisms, underlying rubble is therefore structurally underrepresented. Future studies should employ a higher annotation density (i.e., >10 points per image) and refine methods to decipher this potential limitation to ensure other benthic categories are more readily captured. Observed differences in relative community composition with varying blast ages indicate that benthic communities can exhibit measurable changes following blast fishing disturbances [8].

Since our study represents the first assessment of major benthic genera in Mikindani Bay, it is difficult to define what a fully recovered reef looks like for this region. Although recovery is not guaranteed, it is essential to limit preventable anthropogenic-induced disturbances that hinder the meaningful recovery of coral reef ecosystems. An ecosystem service that is negatively restrained from providing such resources has cascading impacts on the coastal communities that inhabit these regions [63,64]. Coral reefs have been shown to have variable timeframes to recover to pre-disturbance levels, which is dependent on the type of perturbation. However, during these variable recovery timeframes, there are considerable impacts on the livelihoods of coastal inhabitants, and preventing this destructive fishing practice entirely would be greatly beneficial to affected marine ecosystems and local communities [10,23,40]. Management efforts and associated resources should therefore be allocated towards robust enforcement against this destructive fishing practice and rehabilitation initiatives to protect remaining benthic communities and strengthen natural ecosystem resilience.

## 5. Conclusions

Coral reefs affected by blast fishing, a practice that results in the massive deterioration of the marine environment’s overall health, can recover. However, the degree of recovery depends on ecological influences and the frequency of disturbances [6]. Considering ongoing efforts to combat destructive blast fishing in Tanzania and globally, our results emphasize the importance of active enforcement of their goals: protecting and maintaining biodiversity. This requires proactive legislative measures, adequate funding, and long-term monitoring.

Our findings additionally highlight the importance of considering multiple measurements of ecological change, such that aggregate estimates of habitat cover may obscure underlying shifts in community composition. This has equally important impacts on the structure and function of coral reef ecosystems and should be taken into account in monitoring and management efforts. Recovery following blast fishing is a multi-decadal process [8], and this process has the potential to yield ecological communities that differ in structure from pre-disturbed reefs. Conservation management plans should establish realistic recovery targets and acknowledge that reefs recovering from disturbances may function differently, and management plans must adapt accordingly.

## Figures and Tables

**Table 1:** PERMANOVA results showing differences in major benthic genera across transects, sites, survey period, and blast age. Sites and transects were nested by blast age, indicated by parentheses around ‘Blast’. Blast age and survey period were treated as fixed factors, with sites nested within blast age and transects nested within sites.

Source	df	Pseudo-F	% Variance Explained
Blast	2	14.763	25
Survey	3	10.805	5
Site-(Blast)	3	2.827	2
Blast × Survey	6	4.557	6
Transect(Site-(Blast))	6	5.679	2
Site-(Blast) × Survey	9	1.765	1
Transect(Site-(Blast)) × Survey	18	2.027	2

**Table 2:** PERMDISP analysis comparing variability in major benthic genera amongst the three categories of sites.

Deviation from Centroid				
F: 729.77	df1: 2	df2: 1576		P(perm): 0.001
Pairwise Comparisons				
Groups			t	P(perm)
Blast <5 years, Blast >10 years			4.732	0.001
Blast <5 years, Blast 5–10 years			29	0.001
Blast >10 years, Blast 5–10 years			26.584	0.001

## Data Availability

All 2534 images taken during field surveys and data utilized for analysis can be accessed at the following link: https://coralnet.ucsd.edu/source/3681/ (accessed on 29 January 2024).

## Supplementary Materials

The following supporting information can be downloaded at: https://www.mdpi.com/article/10.3390/biology15181584/s1, Table S1. 48 transects and corresponding information for Site, Survey Period, Blast Age, Number of Photos per Survey and Survey Date. Table S2. All 48 taxonomic labels within CoralNet’s Labelset, consisting of Name, Short Code, and corresponding Functional Group. Table S3. PERMANOVA results showing differences in major benthic groups, Percent cover, Taxonomic richness and Shannon diversity across transects, sites, survey period, and blast age. Sites and transects were nested by blast age, indicated by parentheses around ‘Blast’. Blast age and survey period were treated as fixed factors, with sites nested within blast age and transects nested within sites. Table S4. SIMPER analysis reveals major benthic taxon responsible for differences between sites with varying blast ages.

## Abbreviations

The following abbreviations are used in this manuscript:

MDS	Metric Multidimensional Scaling
PERMANOVA	Permutational Multivariate Analysis of Variance
PERMDISP	Permutational Multivariate Analysis of Dispersion
