# Supplementary material for: Benthic Community Composition of Coral Reefs Is Associated with the Time Since Last Blast Fishing Event
Source: Biology (Basel). 2026 Sep 9;15(18):1584. doi: 10.3390/biology15181584 (PMC13604582; doi:10.3390/biology15181584)
Supplement: Supplementary file 1 [file biology-15-01584-s001.zip › biology-4450956-supplementary.pdf]

Supplemental Materials for Leitman et al. 2026 – Benthic Community Composition of Coral Reefs Is Associated with the Time Since Last Blast Fishing Event

Table S1. 48 transects and corresponding information for Site, Survey Period, Blast Age, Number of Photos per Survey and Survey Date.

Site	Survey Period	Blast Age	Number of Photos per Survey	Survey Date
Msangamkuu	1	Blast <5 years	51	12/8/2022
Msangamkuu	2	Blast <5 years	53	7/15/2023
Msangamkuu	3	Blast <5 years	53	1/1/2024
Msangamkuu	4	Blast <5 years	53	9/1/2024
Msangamkuu	1	Blast <5 years	51	12/8/2022
Msangamkuu	2	Blast <5 years	51	7/15/2023
Msangamkuu	3	Blast <5 years	53	1/1/2024
Msangamkuu	4	Blast <5 years	53	9/1/2024
Mwamba Dadi	1	Blast <5 years	51	12/5/2022
Mwamba Dadi	2	Blast <5 years	52	7/15/2023
Mwamba Dadi	3	Blast <5 years	56	1/1/2024
Mwamba Dadi	4	Blast <5 years	53	9/1/2024
Mwamba Dadi	1	Blast <5 years	51	12/5/2022
Mwamba Dadi	2	Blast <5 years	52	7/15/2023
Mwamba Dadi	3	Blast <5 years	57	1/1/2024
Mwamba Dadi	4	Blast <5 years	53	9/1/2024
Mkumgu	1	Blast 5-10 years	51	12/7/2022
Mkumgu	2	Blast 5-10 years	52	7/15/2023
Mkumgu	3	Blast 5-10 years	53	1/1/2024
Mkumgu	4	Blast 5-10 years	54	9/1/2024
Mkumgu	1	Blast 5-10 years	51	12/7/2022
Mkumgu	2	Blast 5-10 years	52	7/15/2023
Mkumgu	3	Blast 5-10 years	54	1/1/2024
Mkumgu	4	Blast 5-10 years	53	9/1/2024
Naumbo	1	Blast 5-10 years	57	12/7/2022
Naumbo	2	Blast 5-10 years	53	7/15/2023
Naumbo	3	Blast 5-10 years	53	1/1/2024
Naumbo	4	Blast 5-10 years	56	9/1/2024
Naumbo	1	Blast 5-10 years	51	12/7/2022
Naumbo	2	Blast 5-10 years	53	7/15/2023
Naumbo	3	Blast 5-10 years	53	1/1/2024
Naumbo	4	Blast 5-10 years	53	9/1/2024
Ribunda	1	Blast >10 years	51	12/8/2022
Ribunda	2	Blast >10 years	52	7/15/2023

Ribunda	3	Blast >10 years	53	1/1/2024
Ribunda	4	Blast >10 years	53	9/1/2024
Ribunda	1	Blast >10 years	51	12/8/2022
Ribunda	2	Blast >10 years	52	7/15/2023
Ribunda	3	Blast >10 years	57	1/1/2024
Ribunda	4	Blast >10 years	53	9/1/2024
Shangani	1	Blast >10 years	51	12/5/2022
Shangani	2	Blast >10 years	52	7/15/2023
Shangani	3	Blast >10 years	56	1/1/2024
Shangani	4	Blast >10 years	53	9/1/2024
Shangani	1	Blast >10 years	51	12/5/2022
Shangani	2	Blast >10 years	51	7/15/2023
Shangani	3	Blast >10 years	53	1/1/2024
Shangani	4	Blast >10 years	53	9/1/2024
**Total Number of Photos Analyzed in CoralNet**			2,534	

Table S2. All 48 taxonomic labels within CoralNet's Labelset, consisting of Name, Short Code, and corresponding Functional Group.

Name	Short Code	Functional Group
Acropora (branching)	Acr_bra	Hard coral
Bleached Hard Coral	B_HC	Hard coral
Colpophyllia natans	CoNat	Hard coral
Diploastrea	Diplo	Hard coral
Dipsastraea	Dipsa	Hard coral
Echinopora	Echinopora	Hard coral
Euphyllia	Euphyllia	Hard coral
Galaxea	Galaxea	Hard coral
Goniastrea	Goniastrea	Hard coral
Halomitra	Hal	Hard coral
Hard coral	HC	Hard coral
Herpolitha	Herpo	Hard coral
Leptoria	Leptoria	Hard coral
Lithophyllon	Litho	Hard coral
Lobophyllia	Lobophyl	Hard coral
Montipora	Monti	Hard coral
Oxypora	Oxyp	Hard coral
Physogyra	Phys	Hard coral
Pocillopora	Pocill	Hard coral
POLYPHYLLIA	POLPHIL	Hard coral
Porites	Porites	Hard coral

Recent Dead Coral	RD_HC	Hard coral
Stephanocoenia intersepta	StephInt	Hard coral
Anthelia	anthe	Other Invertebrates
Condylactis gigantea	CONGI	Other Invertebrates
Diadema	Dia_D	Other Invertebrates
Didemnum molle	DidM	Other Invertebrates
Eunicea spp.	EUNSPP	Other Invertebrates
Linckia sp.	LinSp	Other Invertebrates
Millepora	Millepora	Other Invertebrates
Fire Weed Hydroid	P_ach	Other Invertebrates
Pearsonothuria	Pearson	Other Invertebrates
Radianthus magnifica	Rmag	Other Invertebrates
Sclerophytum	Scle	Other Invertebrates
Sponge	SP	Other Invertebrates
Xenia	Xeni	Other Invertebrates
Sediment	Sediment	Soft Substrate
Rock	Rock	Hard Substrate
Broken coral rubble	C-Rubble	Other
Non-Bio	Non-Bio	Other
Shadow	SHAD	Other
Algae	Algae	Algae
Dictyota	Dictyota	Algae
Galaxaura	Galaxaura	Algae
Hypnea	Hyp	Algae
Padina	Padina	Algae
Sargassum	Sargassum	Algae
Turbinaria (algae)	Turbin	Algae

Table S3. PERMANOVA results showing differences in major benthic groups, Percent cover, Taxonomic richness and Shannon diversity across transects, sites, survey period, and blast age. Sites and transects were nested by blast age, indicated by parentheses around 'Blast'. Blast age and survey period were treated as fixed factors, with sites nested within blast age and transects nested within sites.

	**Percent cover**								**Taxonomic Richness**		**Shannon Diversity**	
	Biological		Algae		Hard coral		Other invertebrates					
Source	Pseudo-F	Probability	Pseudo-F	Probability	Pseudo-F	Probability	Pseudo-F	Probability	Pseudo-F	Probability	Pseudo-F	Probability
Blast	53.963	n.s.	17.161	n.s.	42.941	n.s.	1.3934	n.s.	0.55311	n.s.	0.74184	n.s.
Survey	25.102	0.0002	35.485	0.002	4.3517	0.03	0.88844	n.s.	0.83249	n.s.	1.0093	n.s.
Site(Blast)	0.81836	n.s.	1.931	n.s.	2.6863	n.s.	4.3133	0.0588	1.8891	n.s.	1.8552	n.s.
Blast x Survey	15.107	0.0006	14.857	0.001	0.84556	n.s.	0.78976	n.s.	0.38505	n.s.	0.45363	n.s.
Transect(Site(Blast))	6.9477	0.0001	2.1238	0.043	5.0444	0.001	4.2387	0.0002	4.1256	0.0007	4.3545	0.0001
Site(Blast) x Survey	0.8663	n.s.	3.4575	0.005	1.4016	n.s.	0.87709	n.s.	2.0129	n.s.	1.7612	n.s.
Transect(Site(Blast)) x Survey	0.94215	n.s.	1.7908	0.02	0.85264	n.s.	1.3695	n.s.	0.90415	n.s.	0.99248	n.s.

Table S4. SIMPER analysis reveals major benthic taxon responsible for differences between sites with varying blast ages.

Groups: Blast <5 Years & Blast >10 Years

Average Dissimilarity = 89.76

Taxon	Blast <5 Years Avg. Abundance	Blast >10 Years Avg. Abundance	Avg. Diss	Diss/SD	Contribution %	Cumulative %
Padina	0.33	0.83	20.91	0.82	23.3	23.3
Porites	0.46	0.19	13.24	0.63	14.75	38.04
Acropora	0.23	0.38	12.2	0.57	13.59	51.64
Euphyllia	0.25	0.26	10.31	0.52	11.48	63.12
Xenia	0.12	0.37	10.03	0.49	11.18	74.3

Groups: Blast <5 Years & Blast 5-10 Years

Average Dissimilarity = 92.30

Taxon	Blast <5 Years Avg. Abundance	Blast 5-10 Years Avg. Abundance	Avg. Diss	Diss/SD	Contribution %	Cumulative %
Euphyllia	0.25	2.88	49.89	1.97	54.05	54.05
Porites	0.46	0.08	9.78	0.57	10.6	64.65
Padina	0.33	0.03	7.03	0.47	7.62	72.27

Groups: Blast >10 Years & Blast 5-10 Years

Average Dissimilarity = 91.20

Taxon	Blast >10 Years Avg. Abundance	Blast 5-10 Years Avg. Abundance	Avg. Diss	Diss/SD	Contribution %	Cumulative %
Euphyllia	0.26	2.88	47.33	1.92	51.9	51.9
Padina	0.83	0.03	14.48	0.68	15.88	67.78
Acropora	0.38	0.02	6.87	0.43	7.53	75.31
